# Editorial: Involvements of TRP Channels and Oxidative Stress in Pain

**DOI:** 10.3389/fphys.2018.01084

**Published:** 2018-08-08

**Authors:** Cristina Carrasco, Mustafa Naziroglu, Laszlo Pecze, José A. Pariente

**Affiliations:** ^1^Department of Physiology, Faculty of Sciences, University of Extremadura, Badajoz, Spain; ^2^Neuroscience Research Center, Suleyman Demirel University, Isparta, Turkey; ^3^Department of Medicine, Faculty of Sciences, University of Fribourg, Fribourg, Switzerland

**Keywords:** TRP channels, oxidative stress, pain, nervous system, nociception

Defined as an unpleasant sensory and emotional experience associated with actual or potential tissue damage, pain impairs the quality of life of millions of people worldwide suffering from a wide range of diseases. Thus, efficient pain relief is a socioeconomic priority at the present time. Significant research efforts are being made by the international scientific community to determine the mechanisms underlying nociception, that is, the transmission and integration of noxious stimuli by our nervous system. Growing evidence points to a complex network including oxidative and nitrosative stress, inflammatory response and cation signaling. In this sense, transient receptor potential (TRP) channels have attracted researchers' attention. Discovered in the photoreceptor cells of Drosophila flies, TRP superfamily is presented in different species and grouped into seven subfamilies: TRPA, TRPC, TRPM, TRPML, TRPP, TRPV, and TPRN. The activation of these channels depends on a number of different physical or chemical stimuli. In mammals, 28 isoforms have been identified. Expression levels are very different in tissues and cells, mediating a myriad of processes in our organism, such as sensory physiology. Last years, it has been observed that the expression levels of four TRP channels (TRPA1, TRPM2, TRPV1, and TRPV4) are high in neurons related to nociception, including dorsal root ganglia (DRG) and trigeminal ganglia neurons. In the next decades, all this intense research on the involvements of TRP channels and oxidative stress in pain could provide essential information for the design of individual and rational treatment strategies for pain relief.

As an introduction to this interesting topic, Jardín et al. propose an update review focused on the function of TRPs in the transduction of noxious sensation, especially TRPV1 and TRPA1, and the current knowledge about differential expression and sensitivity in mammals. Moreover, as many researchers have pointed out, there is a functional link between these two channels. It has been observed that some TRPV1-positive neurons co-express the TRPA1 channel; in addition, its activities are closely modulated by TRPV1 channel. In this sense, the research published by Masuoka et al. in this special issue describes that the activities of TRPV1 channel are also modulated by the presence of TRPA1 channel in primary sensory neurons. Thus, this kind of bidirectional link between specific TRP channels activities is another interesting issue which should be further investigated in the future. Finally, we must not forget that research in other species can shed light on intramolecular mechanisms underlying the functioning of TRP channels. For that reason, Kühn et al. offer us a fascinating vision of the TRPM2 channel in both humans and sea anemone *Nematostella vectensis*. As authors reflect, this particular chanzyme co-activated by intracelullar ADP-ribose and Ca^2+^ has evolved from one gene in a strikingly divergent manner but also has gained analogous functional properties in both species.

Turning to mammals, a growing number of evidences suggest that oxidative stress and TRP channels are involved in noxious sensation. Firstly, different types of pain must be distinguished, such as neuropathic pain (NP). As discussed by Carrasco et al., the incidence of this condition may increase in next years and become to a worldwide public health problem. Current knowledge points out mitochondrial dysfunction induced by nitro-oxidative stress, inflammatory signals, and the overload in intracellular calcium ion as possible underlying mechanisms. Among other chronic diseases, NP is present in cancer patients following treatment with chemotherapeutic agents, mainly oxaliplatin, what is known as chemotherapy-induced peripheral pain (CIPP). Naziroglu and Braidy summarize the scientific evidence related to five temperature-regulated TRP channels (TRPA1, TRPM8, TRPV1, TRPV2, and TRPV4) as novel targets for treating this hardly bearable condition that may persist from months to years following cessation of treatment. Focusing on CIPP caused by oxalipatin, this special issue also contains an experimental research performed by Miyake et al. This interesting study clarifies how this noxious chemotherapeutic agent can act differently on TRPA1 channel, depending on the dose. In addition, results showed that ROS-mediated TRPA1 activation may be a common mechanism in the CIPP caused by low and high oxaliplatin concentrations. Likewise, Uslusoy et al. have observed that increased mitochondrial ROS levels, as well as excessive Ca^2+^ entry and apoptosis, are involved in NP caused by sciatic nerve injury (SNI). As authors demonstrated, this condition can be experimentally alleviated in rats by *Hypericum perforatum* treatment. Thus, the vegetal extract seems to act through inhibition of TRPM2 and TRPV1 channels in sciatic nerve and DRG neurons of SNI-induced rats. Furthermore, the Research Topic also collects experimental works about other pain modalities. The study performed by Sandoval et al. showed the interconnection between Cdk5 activation and ROS production by NOX1 and NOX2/NADPH oxidase complexes during inflammatory pain. Moreover, oxidative stress has been observed to be involved in urinary bladder disorders, particularly, mediating the activation of TRPA1. Although this channel seems to be responsible for urinary bladder abnormalities and hyperalgesia in acute cystitis, its implication in chronic bladder inflammation is less clear. In their work, Oyama et al. provide evidence supporting that, as suspected, TRPA1 might not play a major role in the physiopathology of long-lasting cystitis.

In summary, this research field promises to shed light on this intricated matrix linking oxidative stress, calcium signaling (via TRP channels) and inflammatory signals in different pain modalities. In such a way, all this intense research activity will enable us to design individual and rational treatment strategies for pain relief. In fact, molecular neurosurgery appears as one the future medical possibilities. As explained by Pecze et al., some molecular agents can be used as nano-surgery scalpels, which selectively would remove neurons responsible for noxious sensation. Thus, resiniferatoxin has recently entered to phase II clinical trial, demonstrating its safety and efficay in the removal of specific TRPV1+ inflammatory pain-sensing neurons. This new medical concept could revolutionize not only the treatment of morphine-insensitive pain conditions but also the cancer research.

In this research topic issue, 61 authors contributed 10 articles (6 original research and 4 reviews) from different countries in the world (Spain, Turkey, Chile, USA, Australia, Japan, Germany, Switzerland, and Hungary). This multidisciplinary approach (Physiology, Neuroscience, Geroscience, Pharmacology, Chemistry, Medicine, and Biophysics) gives the special issue to a comprehensive analytical insight, discussing current understanding and offering new experimental data about the involvements of TRP channels and oxidative stress in pain. We hope this compilation provides a knowledge base for future researchers of this promising research area.

## Author contributions

CC wrote the editorial. MN, LP, and JP critically revised the work and approved its version to be submitted.

### Conflict of interest statement

The authors declare that the research was conducted in the absence of any commercial or financial relationships that could be construed as a potential conflict of interest.

